# Essential and toxic metals in animal bone broths

**DOI:** 10.1080/16546628.2017.1347478

**Published:** 2017-07-18

**Authors:** Der-jen Hsu, Chia-wei Lee, Wei-choung Tsai, Yeh-chung Chien

**Affiliations:** ^a^ Department of Safety, Health and Environmental Engineering, National Kaohsiung First University of Science and Technology, Kaohsiung, Taiwan; ^b^ Graduate Master Program in Safety, Health and Environmental Engineering, Department of Safety, Health and Environmental Engineering, National Yunlin University of Science and Technology, Yunlin, Taiwan; ^c^ Department of Safety, Health and Environmental Engineering, National Yunlin University of Science and Technology, Yunlin, Taiwan

**Keywords:** Lead, calcium, magnesium, soup, health risk

## Abstract

**Background:** This investigation examines the extraction of metals from animal bones into broth, and assesses whether bone broths are good sources of essential metals and the risks associated with the consumption of toxic metals.

**Method:**Three sets of controlled experiments were performed to study the factors (cooking time, acidity, bone type and animal species) that influence metal extractions. Three types of animal bone broth-based foods were also tested.

**Results:** Reducing the broth pH from 8.38 to 5.32 significantly (*p* < 0.05) increased Ca and Mg extraction by factors of 17.4 and 15.3, respectively. A long cooking time, > 8 h, yielded significantly higher (*p* < 0.05) Ca and Mg extraction than shorter cooking times. The extraction characteristics of metals, particularly Ca, Mg, Cu and Al, from the leg and rib bones differed. The between-species variations in extraction were larger than those of within-species.

**Conclusions:**The Ca and Mg levels in home-made or commercial broth/soup were found not to exceed low tenths of milligram per serving, or <5% of the daily recommended levels. The risks that are associated with the ingestion of heavy metals such as Pb and Cd in broth are minimal because the levels were in the ranges of a few μg per serving.

The health benefits of bone broth (or soup) have been long perceived [1], but only a decade ago was the remedial effect of bone broth scientifically evaluated [2]. For instance, the generally believed curing effect of chicken soup against symptomatic upper respiratory tract infection has been found to follow from an increase in nasal mucus velocity [3] or its mild anti-inflammatory effect [4]. More recently, bone broth has been increasingly recommended as part of the diets for gut and psychology syndrome (GAPS) patients, such as those with autism and attention-deficit hyperactivity disorder (ADHD) [5].

Others regard bone broths as an important dietary source of essential elements, such as calcium, which is particularly favoured by those who are intolerant to, or cannot access, milk products. For example, in some Asian cultures, the consumption of soup made from soaking chicken or other bones in vinegar has been traditionally prescribed for calcium or iron enrichment, especially during pregnancy and postpartum periods [6–8]. Although dietitians and the media have widely promoted bone broth as a calcium supplement, no or only weak scientific evidence concerning calcium levels therein and preparation methods has been provided.

Animal bones are known to contain trace amounts of toxic metals in addition to minerals. Calcium supplements that are made from bonemeal (finely crushed bone) have a lead level in the range of a few to 10 μg/g, and some even contain cadmium (~2 μg/g) [9–13]. Accordingly, simmered broths of animal bones may br reasonably assumed to contain toxic metals and to therefore cause dietary exposure. However, the presence of toxic metals in bone broth has rarely been studied.

This study investigates the extraction of metals, both essential and toxic, from animal bones into broth, with a view to addressing some of the public concerns about whether bone broths are good sources of nutrient elements and the risks that are associated with the consumption of toxic metals in bone broth/soup.

## Research design and methods

### Study design

Four factors (cooking time, acidity, bone type and animal species) that may influence the extraction of metals from animal bones into broth are considered herein. Accordingly, three sets of control experiments were carried out following the procedure that is described below. Each test (simmering broth) lasted for 12 h, with broth samples were taken at 0.5, 2, 4, 8 and 12 h. Moreover, three animal bone broth-based foods were obtained from local stores to examine the metal levels in the broth, and to evaluate associated health risks or benefits. The broths were analysed for essential (Ca, Mg, Fe, Zn, Cu and Cr) and toxic (Pb, Cd and Al) metals.

### Test procedures

The animal bones in this evaluation were the rear leg (femur) and rib bones of domestic pigs (both white and black) and bovine leg (femur) bones, all of which were purchased from a local meat market. The white and black pigs were raised in Taiwan and typically fed on forage and food waste, respectively, while the bovine bone was imported from Australia, which was the most common source of local supplies.

The leg bones were cut longitudinally to expose bone marrow and increase the area of the contact surfaces. Bones were firstly rinsed in boiling water for two minutes, as is common practice in making bone broths in Taiwan, and then as much fat and meat residue as possible was removed. The treated bone was then weighed (295–345 g, mean of 303 g) and deionized water (1:4, by weight) was used to prepare the broth. The deionized water was first brought to the boil in a glass beaker before the bone was added. Once the water was boiling vigorously, it was reduced to a simmer (95–100°C) and a watch glass was used to cover the top of the beaker to maintain reflux until sampling.

At each sampling time, 0.5, 2, 4, 8 and 12 h, 130 g of the liquid sample was taken and the beaker (including bone and broth) was weighed to estimate the loss of water. Then, deionized water (boiled) was added to recover the original weight, and simmering was continued until the subsequent sampling. After the pH level was measured and the fat removed, each broth sample was stored in an acid-washed glass vial at –25°C for further treatment.

#### Effect of acidity

A pair of leg bones from a single white pig carcass was tested to avoid inter-individual variability. In the experimental group (broth acidified), acidified water that was made by mixing 20 ml of table vinegar with 1 l of deionized (DI) water was utilized to simmer the broths. After each sampling, this acidic water was also used to make up the water that was lost due to cooking. This treatment yielded pH levels of 5–6 throughout. In contrast, in the control group, non-acidified DI water was used to prepare the broths, which had mean pH levels of 8–8.5 throughout the simmering period. The test was run in triplicate.

#### Effect of bone type

To test the potential effects of the bone type on the extraction of metals, bones (both leg and rib) were obtained from a single white pig carcass to control for inter-individual variation. For each test, the leg sample comprised one partial femur bone (longitudinally cut) while the rib sample comprised three bone pieces in order to have the comparable test weights. Acidified DI water was used in the preparation and to make up the water that was lost during cooking to increase the amounts of metal extraction to improve analytical sensitivity. The test was run in triplicate.

#### Effect of animal species/strain

To test between- and within-species variation in the extraction of metals, leg bones of white pigs (hybrid of Landrace/Yorkshire/Duroc, ~6 months old), black pigs (hybrid of Taoyuan/Duroc, ~8 months old) and bovines (Angus, ~24 months old) were obtained and tested, as described above. These were readily available from the meat market, but the genders of the test animals were unknown. Acidified DI water was used in preparing the broth and to make up for the lost water from the broths. The test was run in triplicate.

### Collections of commercially available bone broth-based foods

Three animal bone broth-based street foods were obtained from local stores in Douliu city (central Taiwan) to evaluate the levels of essential and toxic metals in broths, and the health risks that would be associated with their ingestion. These foods included pork rib chops that had been stewed in Chinese medicinal herb broth (PR, *n* = 7), beef noodle (Chinese-style noodle with stewed beef in bovine bone broth, BN, *n* = 6) and tonkotsu ramen (Japanese-style noodle in pork bone broth, TR, *n* = 6), all of which are readily available. For simplicity, only the liquid portion (broth) of the foods was assessed. After the samples were cool, the soups were filtered using a 20-mesh gauze; the volume and pH level were recorded, and any grease was removed. The remaining contents were acidified by adding 3 ml of diluted (1:1) nitric acid (~67%, Fisher) per litre of broth, and kept at 4°C until analysis.

### Sample metal analysis

The analysis of metals followed the established method with minor modifications to suit the current matrix. The samples (100 ml) were firstly filtered using fiber glass paper (1 μm pore size) to remove residues. Then three ml of diluted (1:1) hydrochloric acid (~37%, trace metal grade, Fisher) and 6 ml of diluted (1:1) nitric acid (~67%, trace metal grade, Fisher) were added. The mixture was heated gently (85°C) on a hotplate for ~3 h until its volume had been reduced to ~20 ml. The temperature was then increased to 95°C, and a watch glass was placed on top of the mixture to allow refluxing for another 30 min. The residue was diluted to 50 ml, filtered using a 0.45-μm pore size filter disk and stored at 4°C until analysis. The instrument is calibrated against certified multi-element standards. The spiked recoveries of Pb, Cd, Cr, Cu, Fe, Zn, Al, Mg and Ca that were achieved by this procedure were 105.8%, 96.5%, 98.9%, 99.6%, 107.8%, 86.2%, 109.3%, 94.3% and 94.6%, respectively.

Macro metals (Ca, Mg, Fe, Al and Zn) in the samples were analysed using an inductively coupled plasma optical emission spectrometer (ICP-OES, model: PERKIN ELMER Optima 5100 DV), while low-level metals (Pb, Cd, Cu and Cr) were analysed using an ICP–mass spectrometer (model: PERKIN ELMER ELAN DRCII). The method detection limits of ICP-MS with the used protocol for Pb, Cd, Cr and Cu were 0.21, 0.17, 0.27 and 0.24 ppb, respectively, and those of ICP-OES for Fe, Zn, Al, Mg and Ca were 10.9, 11.5, 25.2, 72.1 and 14.2 ppb, respectively.

### Data analysis

The results of the three control experiments were expressed as the total mass of each metal in the cooking container per unit weight (wet) of bone (μg/kg or mg/kg), rather than the concentration at each time point, because the preparation of broth followed no fixed recipe. These results were adjusted for the amounts of metals that were removed by sampling or added by the addition of DI water to compensate for losses caused by simmering. The concentrations of the metals in the broths from the commercially available foods were expressed in ppb or ppm.

Data were log-transformed to improve normality due to small sample size. The paired *t*-test was performed to elucidate the differences between pairs of data of two experimental groups. When more than two groups were being compared, ANOVA was utilized, with *post-hoc* Tukey HSD multiple comparison being used to analyse differences between specific sample pairs. Statistical analysis was performed using SPSS, and α level was set at 0.05.

To evaluate the health benefits or risks that are associated with the ingestion of the metals in the broths from commercially available foods, a contribution ratio or hazard quotient approach was used, in which the metal doses, calculated based on an assumed ingested broth volume (mean or maximum) per day and a liquid density of one, were compared with reference doses from either Dietary Reference Intakes (DRIs) from the National Academy of Medicine (for essential elements Ca, Mg, Zn, Fe, Cu and Cr) or the WHO Provisional Tolerable Weekly Intake (PTWI) (for Pb) and ATSDR Minimal Risk Levels (MRLs)(for Al and Cd).

## Results and discussion

Animal bones contain minerals and so simmering them is generally believed to produce a broth that also contains minerals. Studies have demonstrated that calcium and magnesium levels in broth are generally related to cooking time [7], but little is known about the extraction of other elements, including toxic metals, in broths.

### Effect of acidity on extraction of metals

**Figure 1.**
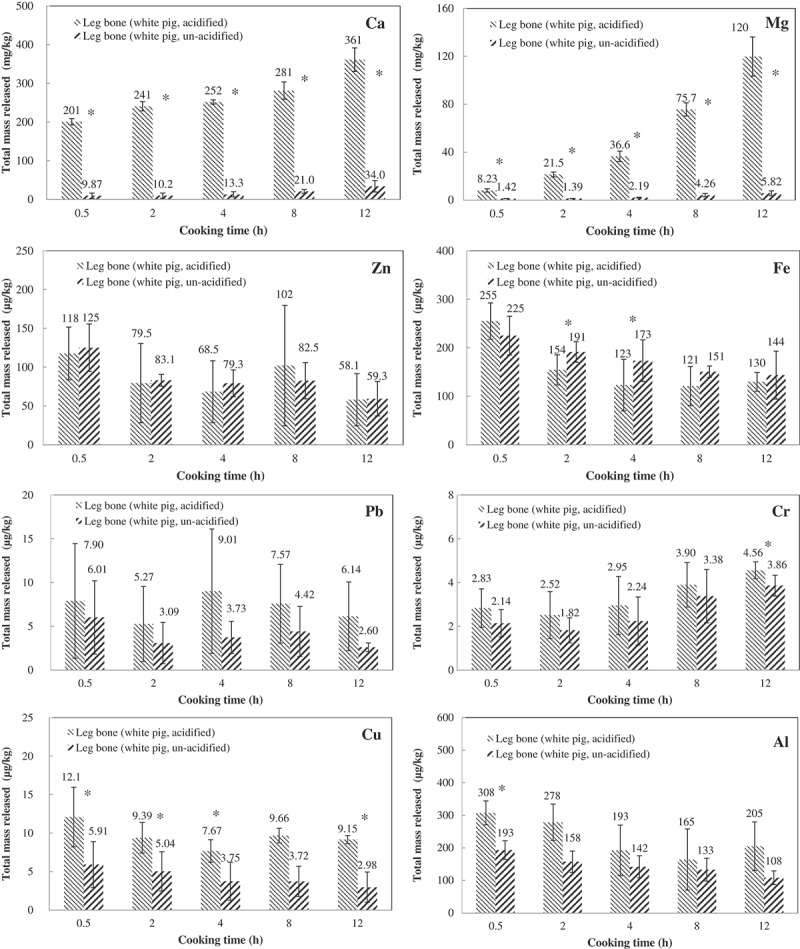
Total amounts released for each metal across different sampling time points between acidified and unacidified broths. The numbers shown are means of triplicate measurement and error bars are standard deviations. The asterisk indicates statistically significant difference (*p* < 0.05, paired *t*-test) between the two broths.

Figure 1 presents the total released/extracted amounts of the metals of interest, normalized to bone weight, in the two test broths, acidified or unacidified, throughout the experimental period. The cadmium levels in all samples were below the current method detection limit and so are not discussed further.

Herein, the addition of acid to the broth increased the amounts of all metals extracted, except for Fe and Zn (Figure 1). The increases were greatest and statistically significant (*p* < 0.05) for calcium and magnesium, with increases of 20.4, 23.6, 18.9, 13.4, 10.6 (mean = 17.4) and 5.8, 15.5, 16.7, 17.8, 20.6 (mean = 15.3) times relative to unacidified broths at 0.5, 2, 4, 8 and 12 hours, respectively. The highest increase was found at the 2nd h for calcium, while for magnesium an increasing trend over time existed.

Several studies have found that increasing the acidity (reducing the pH) increased calcium levels in bone broths. However, findings have been inconsistent. For example, the calcium concentration in a soup that is made of cow vertebra and various vegetables (simmered for 24 h, pH = 4.48) was found to be ~26 times that in a soup without vegetables (pH = 7.06) [7]. Conversely, lowering the pH of a soup that was made from chicken wings (simmered for 2–6 h) from ~7.0 to ~5.7 by adding vinegar caused a small increase, by a factor of 1.4, in calcium concentration [14]. The control broth (unacidified) in the current study had an overall mean pH of 8.38 throughout the test period, while the test broth (with diluted vinegar added in the preparation and used to make up the lost water due to sampling and simmering) had a mean pH of 5.32. Current test results further verified that cooking time and pH significantly affected the calcium concentration in the broth. Hence, a very low pH of the broth might be needed to extract more – say 10–20 times more – calcium and magnesium from bones. Accordingly, the concentrations of calcium and magnesium in traditionally made bone broths are expected to be limited to low tenths of μg/ml under normal cooking conditions.

Similarly, the acidification of the broth significantly (*p* < 0.05) increased the amount of copper extracted by a factor of ~2 throughout, but the increases for lead, chromium and aluminium were smaller, and mostly not significant. In contrast, acidification reduced the extraction of iron, and particularly over 2–4 h, while acidification caused no effect on zinc extraction.

### Effect of bone type on extraction of metals

Long bones such as leg bones are composed of mainly (~80%) dense layers with a high mineral content (known as compact bone), supporting body activities and skeletal mobility, while flat bones such as rib bones have more (50%–75%) loose, spongy layers (known as cancellous bone), which contain bone marrow that forms red blood cells. Nonetheless, spongy bones are always covered by a layer of compact bone for protection. The hard matrix of compact bone (hydroxyapatite) contains crystals of calcium phosphate, calcium carbonate and magnesium salts with collagen fibres that make the bone stronger and somewhat flexible [15,16]. These structural differences in bone potentially influence its mineral composition and, thereby, the extraction of metals therefrom.

**Figure 2.**
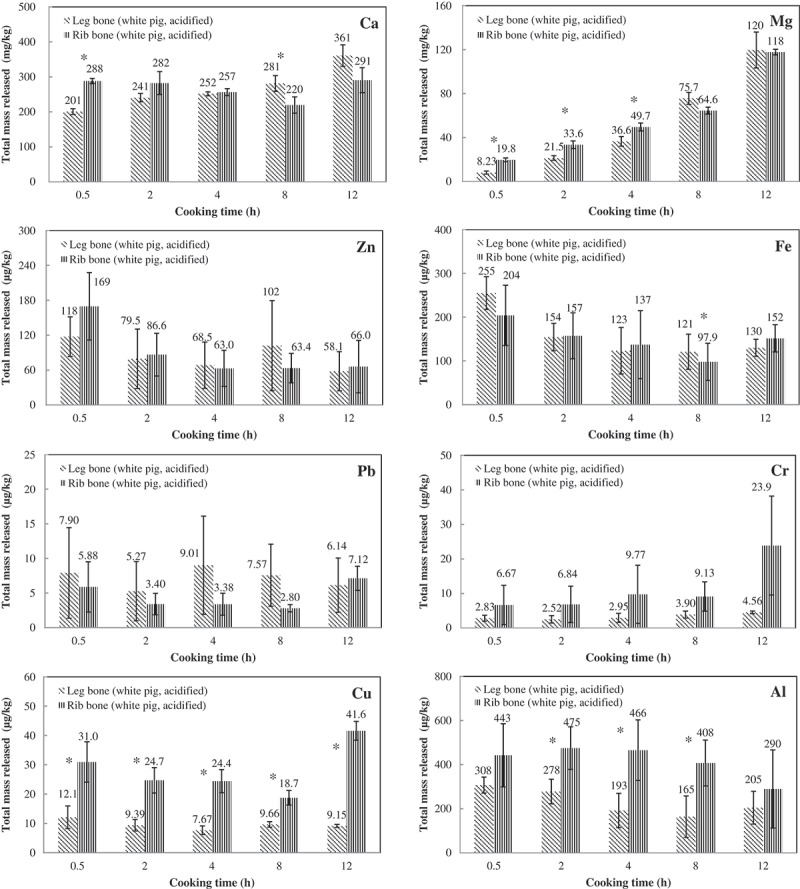
Total amounts extracted for each metal across different sampling time points between the broths made from leg and rib bones. The numbers shown are means of triplicate measurement and error bars are standard deviations. The asterisk indicates statistically significant difference (*p* < 0.05, paired *t*-test) between the two broths.

In this substudy, metal extractions were tested on leg (femur) and rib bones from a single pork carcass. Figure 2 shows the test results throughout the experimental period. The comparison revealed that more (*p* < 0.05) calcium was extracted from the rib bone at first, but that the amounts extracted remained constant thereafter until the end of the test (Figure 2). Conversely, the amount of calcium extracted from the leg bone increased over time, therefore, at the 8th hour and thereafter, the amount extracted exceeded (*p* < 0.05) that extracted from the rib bone. The amounts of magnesium from both broths increased over time, but those from the rib bone were significantly higher (*p* < 0.05) from the beginning until the 8th hour. These discrepancies can be reasonably explained by the facts that the rib sample comprised three bone pieces, which had more contact surfaces [17] for Ca and Mg extraction from the outer compact portion of the bone initially. However, leg bones are mainly composed of mineral matrix and thus can extract Ca and Mg constantly during cooking.

Conversely, the amounts of Cu that were extracted from the rib were significantly higher (*p* < 0.05) than those from the leg bone throughout, but for Al, only amounts extracted at the 2nd, 4th and 8th hour were statistically significantly higher from the rib (*p* < 0.05). The higher level (on average ~3-fold) of Cu that was extracted from the rib than the leg bone may reflect the fact that Cu is important in maintaining the proper function of bone marrow and so is present in a greater quantity in rib (flat) bones [18]. Similarly, an average of 1.9-fold more Al was extracted from the rib bones, which also reflects the structural and functional differences of the bones because a low level of Al has been found to be beneficial to bone mitogenicity [19]. Although not reaching statistically significant level, greater amounts of Cr were extracted from rib than from leg bones. This is compatible with the understanding that the cancellous bone is the most metabolically active type of bone tissue; therefore, its chromium content, which is critical to the metabolism and storage of carbohydrate, fat and protein, can be high [20].

No systemic differences in the amounts of Fe and Zn extracted between the two bone types could be identified. This finding, in conjunction with the earlier discussion that lowering the pH in broths increased the extraction of all metals except Fe and Zn from bones, suggests that the compositions of Fe and Zn in bone are not constant, even though they are crucial for the metabolic processes related to bone formation [21].

Collectively, the extraction characteristics of bones differed between the leg (long) and rib (flat) bones with respect to the content of selected elements, particularly Ca, Mg, Cu and Al.

### Effect of animal species on extractionof metals from bone

Previous studies have examined the extraction of essential elements, mainly calcium, from various animal bone sources, including veal, bovine and even chicken wing, with different cooking treatments [1,7,8,14]. Thus, inter-species comparisons are difficult to perform. This study focuses on inter-species variation in metal extraction by testing leg (femur) bones from two species, pig and bovine, that are commonly used to prepare bone broths. Additionally, white and black pigs were also tested to elucidate intra-species differences.

Table 1 presents the total amounts of metals extracted, normalized to bone weight, in the three test broths throughout the experimental period, statistical test results for the three broths at each time point, and for each broth among various time points. The three bones under consideration did not vary significantly in the amounts of metals extracted, except for copper, iron and magnesium.

**Table 1.** The effect of animal species on the extraction of metals from bone.

		Cooking time (h)						
Metal	Broth	(A) 0.5	(B) 2	(C) 4	(D) 8	(E) 12	*p*-value^a^	Tukey-test^a^
Pb (μg/kg)	(1) Pig (white)	7.90 ± 6.54	5.27 ± 4.28	9.01 ± 7.09	7.57 ± 4.49	6.14 ± 3.92	0.919	–
	(2) Pig (black)	6.55 ± 1.42	4.07 ± 1.50	5.34 ± 2.97	3.86 ± 1.37	4.33 ± 0.73	0.369	–
	(3) Bovine	5.64 ± 4.78	4.42 ± 2.73	3.26 ± 1.59	2.98 ± 1.22	2.53 ± 0.83	0.621	–
	*p*-value^b^	0.847	0.888	0.355	0.185	0.249	–	–
Cr (μg/kg)	(1) Pig (white)	2.83 ± 0.87	2.52 ± 1.07	2.95 ± 1.32	3.90 ± 1.02	4.56 ± 0.38	0.139	–
	(2) Pig (black)	3.36 ± 2.55	3.20 ± 2.11	2.98 ± 1.77	2.80 ± 1.20	3.82 ± 1.44	0.967	–
	(3) Bovine	3.81 ± 1.44	3.74 ± 2.53	3.89 ± 1.46	4.50 ± 1.82	5.55 ± 2.48	0.781	–
	*p*-value	0.799	0.764	0.707	0.375	0.487	–	–
Cu (μg/kg)	(1) Pig (white)	12.1 ± 3.87	9.39 ± 1.98	7.67 ± 1.48	9.66 ± 0.95	9.15 ± 0.52	0.222	–
	(2) Pig (black)	11.4 ± 1.73	11.0 ± 3.48	12.5 ± 2.78	11.7 ± 2.81	9.03 ± 0.38	0.146	–
	(3) Bovine	15.4 ± 8.66	16.2 ± 11.1	13.6 ± 5.05	14.4 ± 3.51	12.8 ± 1.65	0.973	–
	*p*-value	0.466	0.492	0.158	0.172	0.007	–	–
	Paired-comp^b^	–	–	–	–	3 > 1,2	–	–
Fe (μg/kg)	(1) Pig (white)	255 ± 37.4	154 ± 31.2	123 ± 53.1	121 ± 40.2	130 ± 19.4	0.007	A > C,D,E
	(2) Pig (black)	179 ± 52.0	144 ± 5.43	93.6 ± 11.8	99.4 ± 10.3	125 ± 10.5	0.011	A > C,D
	(3) Bovine	197 ± 95.9	173 ± 59.5	169 ± 130	187 ± 33.2	214 ± 43.4	0.956	–
	*p*-value	0.402	0.662	0.556	0.030	0.014	–	–
	Paired-comp	–	–	–	3 > 2	3 > 1,2	–	–
Zn (μg/kg)	(1) Pig (white)	118 ± 34.0	79.5 ± 51.0	68.5 ± 39.7	102 ± 77.3	58.1 ± 33.6	0.600	–
	(2) Pig (black)	130 ± 50.6	91.2 ± 23.1	99.9 ± 35.4	102 ± 64.9	70.0 ± 31.8	0.590	–
	(3) Bovine	132 ± 27.3	122 ± 56.8	83.1 ± 50.7	90.3 ± 38.3	62.1 ± 16.0	0.278	–
	*p*-value	0.890	0.541	0.680	0.966	0.874	–	–
Al (μg/kg)	(1) Pig (white)	308 ± 36.6	278 ± 55.8	193 ± 77.5	165 ± 94.0	205 ± 74.5	0.141	–
	(2) Pig (black)	735 ± 52.3	719 ± 41.5	718 ± 42.0	435 ± 41.0	381 ± 42.0	0.000	A,B,C > D,E
	(3) Bovine	607 ± 476	626 ± 474	639 ± 568	327 ± 156	304 ± 250	0.737	–
	*p*-value	0.232	0.202	0.193	0.057	0.421	–	–
Mg (mg/kg)	(1) Pig (white)	8.23 ± 1.40	21.5 ± 2.07	36.6 ± 4.38	75.7 ± 5.45	120 ± 16.3	0.000	A < C,D,E; B,C < D,E
	(2) Pig (black)	20.0 ± 10.8	31.7 ± 10.4	40.4 ± 2.43	61.7 ± 27.1	115 ± 17.9	0.000	A,B,C,D < E
	(3) Bovine	8.12 ± 4.64	13.8 ± 3.41	23.3 ± 3.47	53.3 ± 15.7	92.7 ± 10.8	0.000	A,B,C < D,E; D < E
	*p*-value	0.125	0.039	0.002	0.379	0.151	–	–
	Paired-comp	–	2 > 3	1, 2 > 3	–	–	–	–
Ca (mg/kg)	(1) Pig (white)	201 ± 8.09	241 ± 11.8	252 ± 5.51	281 ± 22.5	361 ± 30.6	0.000	A,B,C,D < E; A < D
	(2) Pig (black)	240 ± 53.57	252 ± 19.05	226 ± 50.7	276 ± 67.8	439 ± 48.0	0.002	A,B,C,D < E
	(3) Bovine	235 ± 106	274 ± 48.3	343 ± 75.1	371 ± 75.4	444 ± 59.3	0.047	A < E
	*p*-value	0.762	0.448	0.075	0.173	0.134	–	–

^a^ Differences among five (A – E) time groups, tested using ANOVA and *post-hoc* Tukey HSD multiple comparisons; α level is 0.05.

^b^ Differences among three [(1)–(3)] animal groups, tested using ANOVA and *post-hoc* Tukey HSD multiple comparisons.

Bovine-bone broth generally extracted more copper than pig-bone broths, but only significantly (*p* < 0.05) at the 12th hour. Similarly, significantly higher (*p* < 0.05) amounts of iron were extracted in bovine-bone broth than in pig-bone broths following a long period (8th and 12th hours) of simmering.

Conversely, all three broths extracted an increasing amount of magnesium over time, but more (*p* < 0.05) magnesium was initially extracted from black-pig bone than from white-pig or bovine bone. After more than 4 h of cooking, the variation in the amounts of metals extracted from the three bones had diminished and became insignificant.

Bone tissue microstructure differs among mammals. For example, the measured parameters of Haversian canals, Haversian systems and primary osteon vascular canals of femoral compact bone in cows were mostly statistically higher than those of pigs, but large intra-species variability was also noted [22,23]. Bone type, bone portion tested, animal sex and age, and pathological conditions have been found to contribute to such variation [24]. Thereby, variation in the amounts of mineral extracted from bones across and within species is expected but rarely assessed. Herein, within-species (black versus white pig) variation found was generally small and insignificant, while between-species (bovine versus pig) variation was larger and statistically significant on a few metals at some time points (Table 1).

Moreover, mineral-related feed supplements such as clays may also affect the metal loadings in animal body [25], and their impacts on metal distribution in bone warrant further investigations.

### Effect of cooking time on extraction of metals

Studies have shown that the amounts of calcium and magnesium that are extracted from bones generally increase with cooking time [1,7]. The experimental results herein confirm this relationship (Figure 1 and Table 1). Longer cook times, > 8 h, were associated with significantly greater (*p* < 0.05) calcium and magnesium extraction.

Linear regression models were developed using the experimental data to predict of calcium and magnesium concentrations in bone broths that are cooked for up to 12 h. The dependent variable *y* is the total amount of specific metal that is extracted per kilogram of bone (in mg/kg), while the independent variable *x* is the cooking time, in hours. Accordingly, the formula *y* = 2.1281*x* + 6.4153 (*r*^2^ = 0.95, *p* < 0.05) was derived to predict the amount of calcium that is extracted in unacidified broth that is made from the leg bone of a white pig; for magnesium, the equation is *y* = 0.4128*x* + 0.828 (*r*^2^ = 0.98, *p* < 0.05). The non-zero intercept of each formula indicates an appreciable background level, which is compatible with previous findings that minerals can be extracted without cooking/heating [1]. Notably, the predictions, *y*, represent total amounts of metal extracted, so the concentration in the broth can be determined if the actual volume of the broth is known. These models can also be applied to predict the amount of calcium extracted from bovine or black pig bones because the differences in calcium extraction between them were insignificant (Table 1). For broths with vegetables (acidified), a similar model can be derived, based on current data, to predict the amounts of calcium and magnesium extracted when the pH level in broth can be controlled.

The Fe and Al extractions from pig bones were significantly higher at the beginning (0.5 h) than at later periods (Table 1). This could be reasonably anticipated to initially resulting from bone tissues other than minerals, and partially dissolved in fatty portion of the broth as cooking persisted. However, as the metal levels in fat were not measured in this study, additional study is needed to elucidate the metal partitions.

Studies that analyse the concentrations of metals in mineral supplements that are made from finely crushed bone (bonemeal) have improved the understanding of the levels of various metals in animal bones. For example, the concentration ranges of Al, Cr, Cu, Fe, Pb, and Zn in 20 commercial bonemeal supplements samples were 9.34–2040, < 1.0–26.4, 1.10–23.5, 136–1650, 1.5–8.7 and 63.2–156 ppm (μg/g), respectively [13]. These levels were two to three orders of magnitude higher than the extraction ratios found herein, which are in the ranges of parts per billion or micrograms of metal extracted per kilogram of bone (wet weight) (Figure 1). Hence, based on current data, even after 12 h of simmering, only small and stable fractions of the above metals are extracted from bones.

### Health risks and benefits associated with ingestion of broths from commercially available foods

The dissolution of hydroxyapatite (mineral phase of bone) has been found to be positively associated with increasing acidity, contact time, osmolality, temperature, flow rate, surface area and agitation [17]. Because no fixed recipe for preparing bone broth exists, three commercially available animal bone broth-based foods were used to assess the health risks (or benefits) that are associated with the consumption of various metals in broths under realistic exposure conditions.

Table 2 presents the concentrations (mean, standard deviation and maximum) of each metal in the broths from the three commercially available bone broth-based foods, along with the results of a relevant statistical analysis. The analytical results revealed that the broth in each serving of these commercial foods weighed 450–550 g, and that PR (pork rib chops stewed in Chinese medicinal herb broth) had the largest mean weight. The broth of TR (Japanese-style noodle in pork bone broth) had significantly higher (*p* < 0.05) calcium and copper concentrations than those of PR and BN (Chinese-style beef noodle), even though it had a significantly higher (*p* < 0.05) mean pH (6.58). This finding is explained by the cooking times: according to the suppliers of the samples, the TR broth had the longest cooking time, from 10 to 24 h, and was followed in that respect by BN, with a cooking time of > 3 h and PR with a cooking time of 1.5–6 h. Such a long cooking time is essential for TR because the broth, which is made mainly from pork bones, becomes creamy as a result of bone gelatin. The high copper level in the broth may be caused by adding other flat bones, such as scapula, in the preparation of the broths, as discussed earlier. BN broth had significantly higher (*p* < 0.05) iron, zinc and magnesium concentrations than the other two broths, likely because, along with the bovine bones, beef chops are also stewed in the broth for a few hours. This postulation is supported by the findings that large amounts of Fe (23 mg/kg) and Zn (34 mg/kg) have been found in the cattle meat [26].

**Table 2.** Metal concentrations in the broths of the threecommercially availablebone broth-based foods.

Variable	Broth^a^	Mean	SD	Maximum	*p*-value^b^	Tukey-test^b^
Broth weight^c^(g)	PR	555	53.0	639	0.042	PR>BN
	BN	452	90.8	555		
	TR	486	53.1	571		
pH value	PR	5.96	0.26	6.32	0.001	TR>PR TR>BN
	BN	5.61	0.27	5.80		
	TR	6.58	0.50	7.32		
Pb (ppb)	PR	2.63	1.28	4.28	0.232	–
	BN	3.64	3.15	9.39		
	TR	4.27	1.08	5.51		
Cd (ppb)	PR	0.27	0.33	0.89	0.628	–
	BN	0.64	0.64	1.50		
	TR	0.48	0.67	1.68		
Cr (ppb)	PR	4.68	1.96	8.83	0.271	–
	BN	7.49	3.85	14.4		
	TR	6.12	3.61	13.0		
Cu (ppb)	PR	27.9	3.59	30.4	0.002	TR>PR TR>BN
	BN	35.2	6.42	42.4		
	TR	48.7	14.1	72.2		
Fe (ppm)	PR	0.60	0.12	0.81	0.042	BN>TR
	BN	1.14	0.67	2.32		
	TR	0.54	0.25	0.88		
Zn (ppm)	PR	0.16	0.07	0.27	0.033	BN>PR
	BN	0.34	0.19	0.68		
	TR	0.23	0.09	0.34		
Al (ppm)	PR	0.49	0.26	0.83	0.378	–
	BN	0.74	0.23	1.02		
	TR	0.78	1.17	3.16		
Mg (ppm)	PR	24.5	5.50	34.0	0.033	BN>PR BN>TR
	BN	38.6	5.56	44.5		
	TR	35.0	14.3	51.8		
Ca (ppm)	PR	26.1	6.63	34.3	0.000	TR>PR TR>BN
	BN	47.6	14.5	67.7		
	TR	79.9	26.0	111		

^a^PR: pork rib chops stewed in Chinese medicinal herb broth (*n* = 7); BN: Chinese-style noodle with stewed beef in bovine bone broth (*n* = 6); TR: tonkotsu ramen (Japanese-style noodle in pork bone broth (*n* = 6). Only liquid portions (broth) were evaluated.

^b^Tested using ANOVA and *post-hoc* Tukey HSD multiple comparisons; α level is 0.05.

^c^Weight of broth in one commercial serving.

For lead, the overall mean dose for commercial broths was 1.73 μg, based on a mean 500 g serving size. This was compatible with a lead level of 7.01 μg/l found in the broth that had been made from chicken bone. Unfortunately, the method by which the chicken-bone broth was prepared in that study was not described [5].

The results of the risk/benefit analysis (Table 3) indicated that the amounts of essential elements that are consumed in the broths were in the order of tenths of mg (Ca and Mg), hundreds of μg (Fe and Zn), tenths of μg (Cu) and a few μg (Cr) per serving. Consequently, the nutritional values, assessed based on the contribution ratios of ingested dose (per serving) to individual DRI, of calcium, magnesium, iron, copper and zinc, were low, as the mean ratios were generally less than 5%. Chromium dose from broths had higher contribution ratios, with a mean of nearly 10% or ~20% for extreme cases. As an outlier, an iron dose of ~1.3 mg, from the consumption of one serving of BN broth, accounts for 16% of the iron DRI.

**Table 3.** Risks or benefits from ingestion of the metals in the three commercially available bone broths.

Metal	Broth^a^	Ingestion dose (μg/serving)			Hazard quotient or contribution ratio^d^
		Mean	Extreme case^b^	Reference dose^c^ (adult)	
Pb	PR	1.5	2.7	250 μg/day	0.006(0.011)
	BN	1.6	5.2		0.006(0.021)
	TR	2.1	3.1		0.008(0.012)
Cd	PR	0.1	0.6	70 μg/day	0.001(0.009)
	BN	0.3	0.8		0.004(0.011)
	TR	0.2	1.0		0.003(0.014)
Cr	PR	2.6	5.6	35μg/day	0.074(0.160)
	BN	3.4	8.0		0.097(0.229)
	TR	3.0	7.4		0.086(0.211)
Cu	PR	15.5	19.4	900 μg/day	0.017(0.022)
	BN	15.9	23.5		0.018(0.026)
	TR	23.7	41.2		0.034(0.046)
Fe	PR	333	518	8 mg/day	0.026(0.065)
	BN	515	1288		0.064(0.161)
	TR	262	502		0.033(0.063)
Zn	PR	88.8	173	11 mg/day	0.008(0.016)
	BN	154	377		0.014(0.034)
	TR	112	194		0.010(0.018)
Al	PR	272	530	70 mg/day	0.004(0.008)
	BN	334	566		0.005(0.008)
	TR	379	1804		0.005(0.026)
Mg	PR	13,598	21,726	400 mg/day	0.034(0.054)
	BN	17,447	24,698		0.044(0.062)
	TR	17,010	29,578		0.043(0.074)
Ca	PR	14,486	21,918	1000 mg/day	0.014(0.022)
	BN	21,515	37,574		0.022(0.038)
	TR	38,831	63,381		0.039(0.063)

^a^PR: pork rib chops stewed in Chinese medicinal herb broth; BN: Chinese-style noodle with stewed beef in bovine bone broth; TR: tonkotsu ramen (Japanese-style noodle in pork bone broth. Broth only.

^b^Estimated based on maximum broth weight times maximum metal concentrations from Table 2, assuming broth density of 1.

^c^Reference dose = DRIs for essential elements Ca, Mg, Zn, Cu, Cr and Fe; the WHO PTWI for Pb and ATSDR MRLs for Al and Cd.

^d^Hazard quotient or contribution ratio = ingested dose/reference dose; Mean (extreme case).

The levels of these metals were slightly higher than or compatible with those listed for the ‘Soup, stock, beef, home-prepared’ category of the USDA National Nutrient Database, in which the levels of Ca, Mg, Fe and Zn are 8, 7, 0.27, 0.17 mg per cup (240 g), respectively. Nevertheless, the ingredients and their quantities for making such soup were not specified in that database. For commercial foods category, such as Swanson soup (beef broth, low sodium), the Ca, Mg, Fe and Zn contents were 8, 4, 0.17, 1.32 mg per can (413 g), but no data are available for a container (298 g) of Campbell’s soup (chicken broth, low sodium). For comparison, the label on a can of ‘Chef Flavor’ bone broth reports the level of calcium per cup serving as being 4% of the US Recommended Daily Allowance (i.e. 1000 mg/day for adults).

The doses of toxic metals that are ingested in these broths are in the range of a few μg (Pb and Cd) and hundreds of μg (Al) per serving. These levels are lower than the respective reference doses (MRLs) and thus resulting in low hazard quotients (generally less than 0.002). Therefore, the health risks that are associated with the ingestion of toxic metals from one serving of these broths are regarded as minimal, with no interactive effect among them being assumed. Nevertheless, consumption of large volume of prolong-cooked bone broth is not recommended as they may contain high level of oil-based ingredients, e.g. vitamin D, which is derived from fatty bone marrow and may result in hypercalcaemia if overdosed [27].

## Conclusions

Bone broths/soups are important foods owing to their taste, nutrients and even curative effects. Their nutritional values, and particularly calcium levels, have attracted attention, but systemic evaluations of methods of their preparation and the range of calcium concentrations are few, as are the health risks associated with the ingestion of toxic metals such as lead that commonly accompany bone minerals.

Like increasing acidity and simmering time, as demonstrated herein, the dissolution of bone mineral has also been found to be positively associated with many other factors, such as temperature, surface area and agitation. Additionally, variation in bone tissue microstructure and mineral distribution across animal bones also affects the mineral levels available for extraction. Therefore, bone minerals that are extracted in broths may be affected by these factors and so be difficult to standardize. The calcium and magnesium levels in home-made or commercial broth/soup, according to the literature and current study, are no more than low tenths of a milligram per serving. These levels are generally a few percent of the DRIs, and so their contributions to daily Ca and Mg requirements are considered to be small. The heavy metals such as Pb and Cd that are present in commercial broth/soup were found herein to have concentrations in the range of a few micrograms per serving. Thus the hazard quotients are low and so the risks that are associated with ingestion of heavy metals from broth are considered minimal.
